# Curcumin Mitigates Neuro-Inflammation by Modulating Microglia Polarization Through Inhibiting TLR4 Axis Signaling Pathway Following Experimental Subarachnoid Hemorrhage

**DOI:** 10.3389/fnins.2019.01223

**Published:** 2019-11-15

**Authors:** YongYue Gao, Zong Zhuang, Yue Lu, Tao Tao, Yan Zhou, GuangJie Liu, Han Wang, DingDing Zhang, LingYun Wu, HaiBin Dai, Wei Li, ChunHua Hang

**Affiliations:** ^1^Department of Neurosurgery, Nanjing Drum Tower Hospital, The Affiliated Hospital Nanjing University Medicine School, Nanjing, China; ^2^Department of Neurosurgery, Nanjing Drum Tower Hospital, Clinical College of Nanjing Medical University, Nanjing, China; ^3^Department of Neurosurgery, Nanjing Drum Tower Hospital, Clinical Medical College of Southern Medical University, Guangzhou, China

**Keywords:** toll-like receptor 4, subarachnoid hemorrhage, curcumin, neuro-inflammation, microglia polarization

## Abstract

Subarachnoid hemorrhage (SAH) elicits destruction of neuronal cells and neurological function, which is exacerbated by neuro-inflammation in EBI, and toll-like receptor 4 (TLR4) plays an important role in inflammatory cascade via modulation microglia polarization. Curcumin (Cur), as a natural phytochemical compound, has the potential characteristics on anti-inflammatory and microglia phenotype transformation. In this study, we verified the hypothesis curcumin promotes M2 polarization to inhibiting neuro-inflammation, which through suppressing TLR4 signaling pathway after SAH. In *tlr4^–/–^* mice and wild type (WT) subjected to prechiasmatic cistern blood injection, Western blotting, brain water content, neurological score, enzyme-linked immunosorbent assay (ELISA) and terminal deoxynucleotidyl transferase dUTP nick end labeling (TUNEL) staining were performed to investigate the role of TLR4 on neuro-inflammation response and microglia polarization. Curcumin with three different concentrations (50 mg/kg, 100 mg/kg and 200 mg/kg) were injected intraperitoneally (i.p.) at 15 min after SAH. The levels of TLR4, myeloid differentiation factor 88 (MyD88), nuclear factor- κB (NF-κB), Iba-1, CD86, CD206 and pro/anti-inflammation cytokines were measured by Western blotting and immunofluorescence staining at 24 h after SAH. SAH induction increased the protein levels of TLR4, pro-inflammation cytokines and proportion of M1 phenotype. Curcumin with 100 mg/kg treatment dramatically inhibited the release of pro-inflammatory mediators, and elevated the protein levels of anti-inflammatory cytokines and promoted microglia switch to M2. Meanwhile, curcumin treatment also decreased the expressions of TLR4, Myd88 and NF-κB at 24 h post SAH. TLR4 deficiency ameliorated brain water content, neurological deficit and reduced pro-inflammation cytokines after SAH. Moreover, curcumin treatment in *tlr4^–/–^* mice further induced M2 polarization, while had no statistic difference on brain water content and neurological score at 24 h post SAH. Our results indicated that curcumin treatment alleviated neuro-inflammation response through promoting microglia phenotype shift toward M2, and which might inhibiting TLR4/MyD88/NF-κB signaling pathway after SAH.

## Introduction

Subarachnoid hemorrhage (SAH) is a neurologic emergency, accounts for approximately 85%, caused by ruptured aneurysms and has a high rate of morbidity and mortality (van Gijn et al., 2007; Ayling et al., 2016). Despite advances in the understanding of SAH pathophysiological processes, complication of it continue to make substantial contributions to poor clinical outcomes. Recent studies demonstrated that early brain injury (EBI), which occur within 72 h following SAH, regarded as the leading cause of poor prognosis (Cahill and Zhang, 2009; Macdonald, 2014). Among the pathogenesis processes of EBI, acute inflammatory injury is one of the critical factors, which is mainly caused by releases of inflammatory mediators and damage-associated molecular patterns (DAMPs) (Chaudhry et al., 2018; Zheng and Wong, 2019). Therefore, an anti-inflammation strategy has attracted attention in the treatment of SAH.

As the brain resident immune cell, microglia play a double-edged role in neuro-inflammation, which is associated with the functional outcome of SAH patients (Wang et al., 2018; Zheng and Wong, 2019). The activated microglia has two polarized phenotypes under different stimulus after SAH, corresponding to M1 and M2 (Butovsky and Weiner, 2018). The former could secrete numerous pro-inflammatory chemokines, including interleukin-1β (IL-1β), interleukin-6 (IL-6), tumor necrosis factor-α (TNF-α) and inducible nitric oxide synthase (iNOS), while the latter exhibit anti-inflammatory properties and produces transforming growth factor β (TGF-β), interleukin-10 (IL-10), and CD206 (Meng et al., 2016; Ransohoff, 2016). Microglia are principal activated into M1 phenotype and aggravate inflammatory response following SAH (You et al., 2016; Li et al., 2018). Therefore, therapeutic intervene the polarization of microglia may serve as a valid target for SAH treatment.

Toll-like receptors (TLRs), as the transmembrane signal transduction molecules, are able to recognize conserved microbial motifs including lipopolysaccharide (LPS) and endogenous ligands such as high-mobility group box 1 (HMGB1), heat shock proteins (HSP70) (Tahara et al., 2006; Cao et al., 2007). Among these TLRs, TLR4 is one of the most studied receptors, and which could activate its downstream critical adapter protein myeloid differentiation factor 88 (MyD88) and nuclear factor κB (NF-κB), leads to produce pro-inflammatory cytokines and induce inflammatory response (Wang et al., 2013; Lu et al., 2018). Previous studies demonstrated that TLR4 is highly up-regulated and mainly expressed in microglia after brain injury (Zhang et al., 2016; Rosciszewski et al., 2018). In addition, TLR4 deficiency contributes to modulating M1 phenotypic switch to M2, and ameliorates neuro-inflammation after traumatic brain injury (TBI) (Yao et al., 2017). Thus, suppressing TLR4 activation might as a valid target against neuro-inflammation after SAH.

Curcumin, extracted from roots of Curcuma longa, possesses multiple biological activities including anti-inflammatory, anti-oxidant and anti-tumor properties (Zhu et al., 2014; Maiti et al., 2019; Mhillaj et al., 2019). Previous *in vivo* and *in vitro* studies have proven that curcumin has anti-inflammation potential to regulate the releases of different inflammatory cytokines, and cross the blood brain barriers (BBB) with high bioactivity (Zhu et al., 2014). Currently, accumulating studies indicated that curcumin could promote microglia phenotype transformation toward M2 and inhibit microglia-mediated pro-inflammation response in neurodegenerative and ischemic diseases (Liu et al., 2017; Pluta et al., 2018). Thus far, although cumulative findings indicate that multiple mechanisms might be involved in the effect of curcumin on anti-inflammatory (Zhu et al., 2014; Porro et al., 2019; Sinjari et al., 2019), the potential mechanism of curcumin on microglia phenotypic polarization after SAH remains obscure. Therefore, we investigate the effects of curcumin on microglia polarization and the potential mechanism of attenuating SAH-induced neuro-inflammation.

## Materials and Methods

### Animals

In this study, adult male C57BL/6J mice weighing between 25 and 28 g were purchased from the Experimental Animal Center of Drum Tower Hospital (Nanjing, China), and *tlr4^–/–^* mice (C57BL/10Sc NJ, 25–28 g, male) and wild type (WT) mice were purchased from Nanjing Biomedical Research Institute Nanjing University. All mice were fed in a 12-h light/dark cycle room with controlled humidity and temperature (24 ± 0.5°C). All experimental protocols and procedures for this study were approved by the Institutional Animal Care and Use Committee at Drum Tower Hospital and conformed to *the National Institutes of Health (NIH) Guide for the Care and Use of Laboratory Animals*.

### SAH Models

The prechiasmatic cistern SAH models used in this study were established as we previously reported (Lu et al., 2019). Briefly, mice were fixed on a stereotaxic apparatus after inhalation anesthesia with isoflurane (2% in oxygen gas, 300 ml/min). After the disinfection, nearly 1.0 cm midline scalp incision was made and a hole with diameter of 1.0 mm was drilled with 4.5 mm anterior to the bregma through the skull in the midline. One mouse was euthanized as a donor for arterial blood by exposed left ventricular cardiac puncture, and approximately 50 μl arterial blood was injected into the prechiasmatic cistern through the prepared hole with needle kept in this position for at least 2 min to prevent blood backflow or cerebrospinal fluid (CSF) leakage. The drilling point sealed with bone wax and sutured incision sterile. Then, animals were allowed to recover for 45 min after SAH, returned to the cages and maintained at a temperature of 24.0 ± 0.5°C. Sham-operated mice underwent the same procedures, but was injected into equal volume of normal saline solution.

### Experimental Design and Drug Administration

**FIGURE 1:**
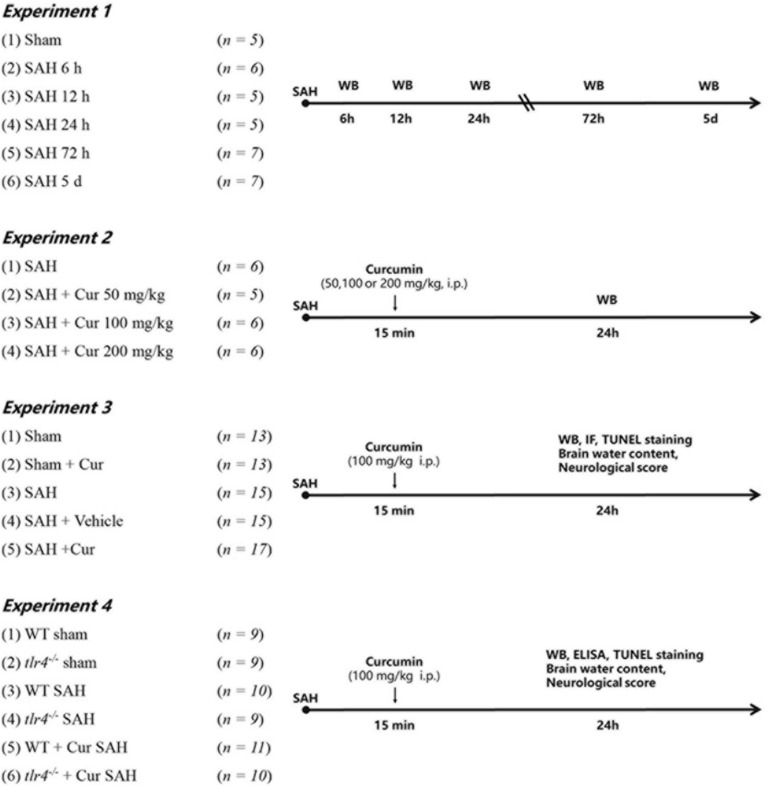
Experimental design of this study. Cur, curcumin; WT, wild type; Vehicle, Saline containing 10% dimethyl sulfoxide (DMSO); WB, Western blotting; IF, immunofluorescence staining; ELISA, enzyme-linked immunosorbent assay.

All mice were randomly assigned to the following experiments as described (Figure 1).

#### Experiment-1

To determine the expression of TLR4 in the cortex of mice after SAH. Mice were randomly assigned to seven groups: Sham group (*n* = 5) and SAH group (6 h, 12 h, 24 h, 72 h, 5 day) (*n* = 5/group); 5 mice of each group were selected randomly for western blot analyses.

#### Experiment-2

To determine the optimal dosage of curcumin after SAH. Mice were divided randomly into four groups: SAH group, SAH + Cur group (50 mg/kg, 100 mg/kg and 200 mg/kg) (*n* = 5/group). Curcumin (Sigma, Saint Louis, MO, United States) with purity more than 96% dissolved in 100 μL saline containing 10% dimethyl sulfoxide (DMSO) and injected intraperitoneally (i.p.) at 15 min post SAH induction according to previous study (Zhu et al., 2014). Based on the expression of TLR4, 100 mg/kg of curcumin was chosen for the next experiments.

#### Experiment-3

To explore the effects of curcumin on anti-inflammation response and potential mechanism on microglial polarization after SAH. Mice were randomly assigned into the five groups: Sham group, Sham + Cur group, SAH group, SAH + Vehicle group, SAH + Cur group. Assessment method including western blot analyses (*n* = 5/group), double immunofluorescence staining and deoxynucleotidyl transferase dUTP nick end labeling (TUNEL) staining (*n* = 3/group), brain water content and neurological score (*n* = 5/group).

#### Experiment-4

To further evaluate the effect of TLR4 on microglia polarization and neuro-inflammation after SAH. The *tlr4^–/–^* and WT mice were randomly assigned to six group: WT Sham group, *tlr4^–/–^* Sham group, WT SAH group, *tlr4^–/–^* SAH group, WT SAH + Cur group, *tlr4^–/–^* SAH + Cur group (*n* = 9 each). Assessment method including western blot analyses and enzyme-linked immunosorbent assay (ELISA) (*n* = 3/group), TUNEL staining (*n* = 3/group), brain water content and neurological score (*n* = 3/group).

### Immunofluorescence Staining

Double-immunofluorescence Staining was conducted as previously described (Lu et al., 2018, 2019). Briefly, brain samples dehydrated with 30% sucrose at least 24 h and tissue sections (8 μm thickness) were cut with a cryostat. The slides were incubated in 5% blocking buffer for 2 h at room temperature, then incubated with rabbit anti-CD86 antibody (1:100) or anti-CD206 antibody (1:100), respectively, overnight at 4°C. Afterward, incubated with another primary antibody, namely mouse anti-Iba-1 (1:50), under similar conditions. Alexa-Fluor 594 goat-rabbit IgG and Alexa-Fluor 488 donkey-mouse IgG were used as the corresponding secondary antibodies for 1 h at the room temperature. Then the slides were stained with 4-diamidino-2-phenylindole (DAPI) for 15 min to show the location of nucleus. The fluorescently stained cells were imaged on an Olympus IX71 inverted microscope system and analyzed using the Image-Pro Plus 6.0 software (Media Cybernetics, Silver Spring, MD, United States).

### Terminal Deoxynucleotidyl Transferase-Mediated dUTP Nick-End Labeling (TUNEL) Staining

For TUNEL staining, brain sections were incubated in 4% paraformaldehyde blocking for 30 min at room temperature, then incubated with rabbit anti-NeuN (1:200) overnight at 4°C. Afterward, Alexa-Fluor 488 goat-rabbit IgG was used as the corresponding secondary antibodies for 1 h at the room temperature. The sections were incubated with the TUNEL reagent (Beyotime Biotechnology, Shanghai, China) for 1 h at 37°C. Then the slides were stained with DAPI for 15 min to show the location of nucleus. The number of TUNEL-positive neurons was regarded as apoptosis index. The fluorescently stained cells were imaged on an Olympus IX71 inverted microscope system and analyzed using the Image-Pro Plus 6.0 software (Media Cybernetics, Silver Spring, MD, United States).

### Western Blot Analysis

Exacted from temporal cortex brain tissue, and total protein concentration of the lysate was determined by the Bradford method using Bradford Protein Assay Kit (Beyotime Biotechnology, Shanghai, China). Equal amounts of proteins were resolved on a 10–12% sodium dodecyl sulfate-polyacrylamide gel electrophoresis (SDS-PAGE) gel and transferred onto polyvinylidene fluoride (PVDF) membrane (Immobilon-P, Millipore, Billerica, MA, United States). The membrane was blocked with 5% non-fat dry milk in TBST (Tris-buffered saline with 0.05% Tween 20) for 2 h at room temperature, and then incubated overnight at 4°C, separately with the appropriate primary antibodies against the specific proteins, IL-1β, IL-6, TNF-α, iNOS, IL-10 and TGF-β (1:200, Santa Cruz Biotechnology, United States), TLR4, Myd88, Iba-1, CD86 and CD206 (1:200, Abcam, Cambridge, United Kingdom), NF-κB p65, p-IκB-α, IκB-α and β-Tubelin (1:1000, Cell Signaling Technology, United States), and β-actin (1:5000, Bioworld Technology, United States) in a blocking buffer. Afterward, the membrane incubated with the secondary antibodies, namely HRP conjugated secondary antibodies (goat; Bioworld Technology, United States, 1:5000) or HRP conjugated secondary antibodies (horse; Cell Signaling Technology, United States, 1:1000) for 2 h at room temperature. Finally, the protein bands were visualized via enhanced chemiluminescence (ECL) (Millipore, Billerica, MA, United States) and exposure to X-ray film. The Western blot results were analyzed using Un-Scan-It 6.1 software (Silk Scientific Inc., Orem, UT, United States).

### Enzyme-Linked Immunosorbent Assay

Brain samples in the temporal cortex were mechanically homogenized and centrifuged at 4°C at 12,000 × *g* for 20 min. The supernatants were collected and determined the total protein concentration. The protein levels of IL-1β, IL-6, TNF-α, and IL-10 were quantified via ELISA assay kits (R&D Systems, Minneapolis, MN, United States), in accordance with the manufacturer’s instructions. The cytokines concentrations of tissue were expressed as picograms per milligram of protein.

### Brain Water Content

Brain water content was measured as previously study (Zhou et al., 2015; Gao et al., 2017). Brains were quickly removed at 24 h post SAH. The brainstem was discarded, while the tissue of left hemisphere cortex and right cortex were harvested, and weighted the wet weight of each cortical tissue, then dried for 72 h at 80°C and the dry weight determined. The percentage of brain water content was calculated as the following formula = [(wet weight − dry weight)/wet weight] × 100%.

### Neurologic Score

The neurological deficits were evaluated at 24 h after SAH as previously described (Table 1; Zhou et al., 2015; Zhang et al., 2016). Briefly, three activity examinations were evaluated, including appetite, activity and deficits. Grading of neurologic deficits was as follows: severe neurologic deficit (scores = 4 – 6), moderate neurologic deficit (scores = 2 – 3), mild neurologic deficit (scores = 1), and no neurologic deficit (scores = 0). All the tests were evaluated by two independent observer who was blind to the treatment conditions. Lower scores represented better neurological function.

**TABLE 1:** Behavior score.

**Category**	**Behavior**	**Score**
Appetite	Finished meal	0
	Left meal unfinished	1
	Scarcely ate	2
Activity	Active, squeaking or standing	0
	Lying down, will stand, and walk with some stimulation	1
	Almost always lying down	2
Deficits	No deficits	0
	Unable to walk because of ataxia or paresis	1
	Impossible to walk and stand because of ataxia and paresis	2

### Statistical Analysis

The SPSS 17.0 software package was used for the statistical analysis. All data are expressed as the mean ± Standard Deviation (SD). Comparisons between two groups were performed using Student’s *t* test and multiple comparisons were performed using a one-way ANOVA followed by Tukey’s test. A *p* value < 0.05 was regarded as statistically significant.

## Results

### Mortality Rate

**FIGURE 2:**
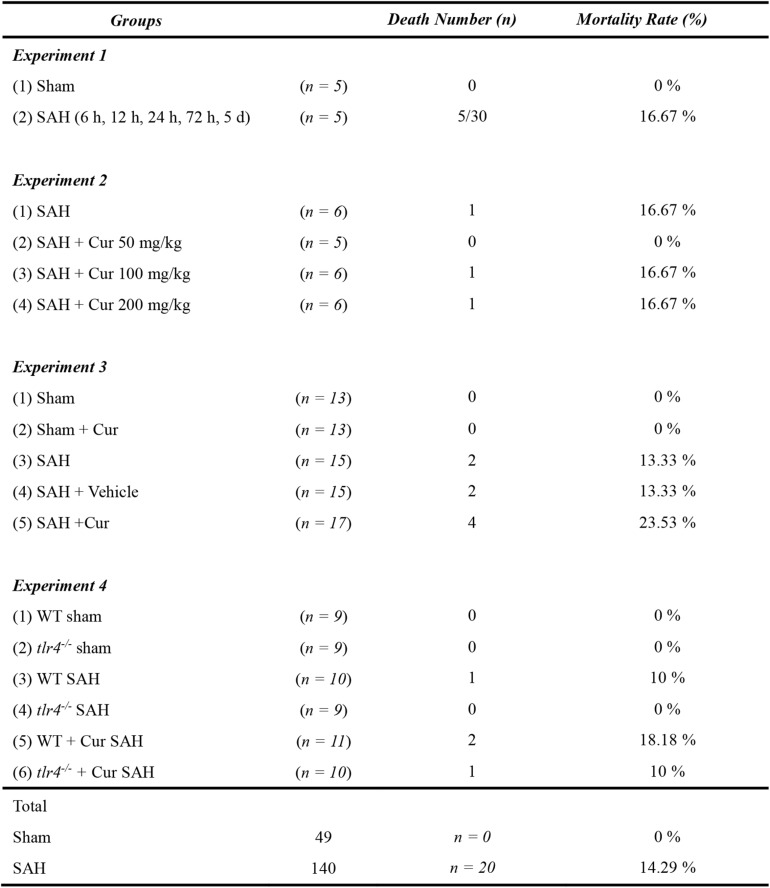
Mortality rate following SAH in mice.

Out of the 189 mice were used in our experiment. 49 mice were part of the Sham group, and 140 mice underwent SAH induction. Twenty mice died after the operation and the overall mortality in SAH group was 14.29%. No animals died in the sham group and sham + Cur group (Figure 2).

### TLR4 Deficiency Attenuated Brain Edema, Neuronal Apoptosis, and Improved Neurological Function Post SAH

**FIGURE 3:**
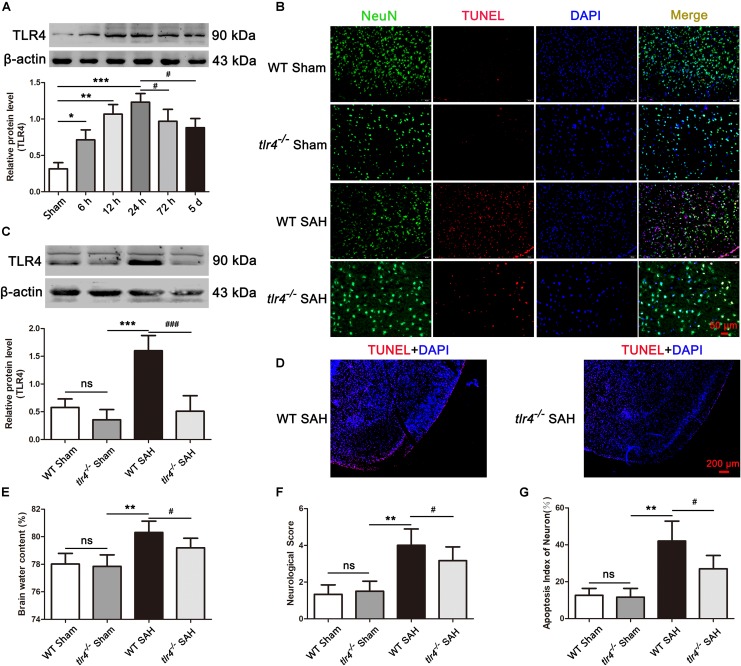
Effects of toll-like receptor 4 (TLR4) deficiency on brain water content, neurological deficit and neural death after SAH. **(A)** SAH induction increased the level of TLR4 and peaked at 24 post SAH. **(B,G)** TLR4 deficiency decreased the TUNEL-positive neurons compared with the wild type (WT) SAH group at 24 h after SAH. **(C)** Western blotting showed that the expression of TLR4 markedly decreased in *tlr4*^–/–^ mice after SAH compared with the WT SAH group. **(D)** The proportion of TUNEL-positive neural cells dramatically decreased in the temporal cortex of *tlr4*^–/–^ mice as compared with WT group after SAH. **(E,F)** TLR4 deficiency alleviated brain edema and neurological deficit at 24 h after SAH. The quantitative data are the mean ± SD [*n* = 5, each; ^∗^*p* < 0.05, ^∗∗^*p* < 0.01, ^∗∗∗^*p* < 0.001 vs. Sham group, *tlr4*^–/–^ Sham group; ^#^*p* < 0.05, ^###^*p* < 0.001 vs. SAH (24 h) group, WT SAH group; ^*ns*^*p* > 0.05]. Scale bars = 50 μm **(B)** and 200 μm **(D)**.

We first identified the expression of TLR4 by Western blot analysis after SAH. As shown in Figure 3A, TLR4 seldom expressed in the sham group, significantly increased at 6 h time point after SAH, and reaching its peak level at 24 h time point. Subsequently, its expression decreased and lasting up to day 5 following SAH. To investigate the role of TLR4 on neuroprotection after SAH, *tlr4^–/–^* mice were used. As shown in Figure 3C, the expression of TLR4 remarkably decreased at 24 h after SAH in *tlr4^–/–^* mice compared with WT mice. Meanwhile, the proportion of TUNEL-positive neuronal cell in temporal cortex were significantly decreased in *tlr4^–/–^* mice as relative to WT mice (Figure 3D). Afterward, brain water content and neurological scores were also measured. Results showed that TLR4 deficiency decreased the brain water content and improved neurological deficits in *tlr4^–/–^* mice after SAH at 24 h as relative to the WT mice (Figures 3E,F). In addition, the number of TUNEL-positive neurons dramatically decreased in *tlr4^–/–^* mice at 24 h after SAH when compared with WT SAH mice (Figures 3B,G).

### Curcumin Inhibited TLR4 Expression and Provided Neuroprotection Post SAH

**FIGURE 4:**
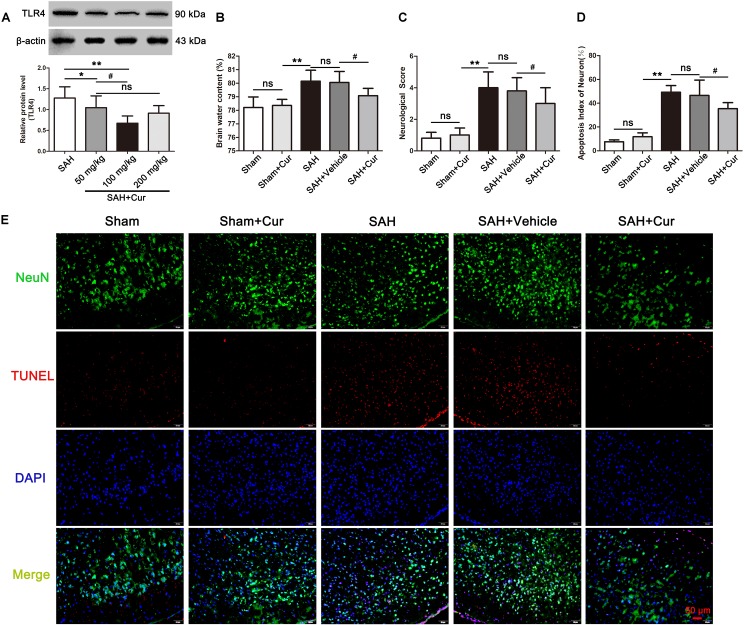
Effects of curcumin (cur) on expression of toll-like receptor 4 (TLR4) and neuroprotection, evaluated by brain water content, neurological score, and TUNEL staining at 24 h after SAH. **(A)** Curcumin with 100 mg/kg significantly reduced the protein level of TLR4 at 24 h post SAH. **(B,C)** Curcumin treatment ameliorated the brain water content and neurological function at 24 h after SAH as compared with the SAH + vehicle group. **(D)** Representative immunofluorescence staining images of TUNEL (NeuN = green, TUNEL = red, DAPI = blue) **(E)** Administration of curcumin remarkably decreased the amount of TUNEL-positive neurons at 24 h after SAH compared with the SAH + vehicle group. The quantitative data are the mean ± SD [*n* = 5, each; ^∗^*p* < 0.05, ^∗∗^*p* < 0.01 vs. SAH group, Sham + Cur group; ^#^*p* < 0.05 vs. SAH + Cur (50 mg/kg) group, SAH + Vehicle group; ^*ns*^*p* > 0.05]. Scale bars = 50 μm.

To assess whether curcumin had effect on TLR4 expression and neuroprotection, three dosages of curcumin (50, 100, and 200 mg/kg) were administrated intraperitoneal 15 min after SAH. The results showed that curcumin treatment markedly decreased the expression of TLR4 at 24 h compared with SAH group. However, the curcumin treated with 100 mg/kg exhibited more effective on reduction TLR4 expression than other two dosages (Figure 4A). Accordingly, we chose 100 mg/kg curcumin for further experiments. Subsequently, brain water content, neurological scores and TUNEL staining were also measured to evaluate the neuroprotection of curcumin. The data showed that administration of curcumin decreased brain water content and improved the neurological function at 24 after SAH, as compared with the SAH + Vehicle group (Figures 4B,C). Meanwhile, curcumin treatment after SAH dramatic reduced the proportion of TUNEL-positive neurons relative to the SAH + Vehicle group at 24 h post SAH (Figures 4D,E).

### Curcumin Treatment Inhibited Microglia Activation and Promoted M2 Polarization Post SAH

**FIGURE 5:**
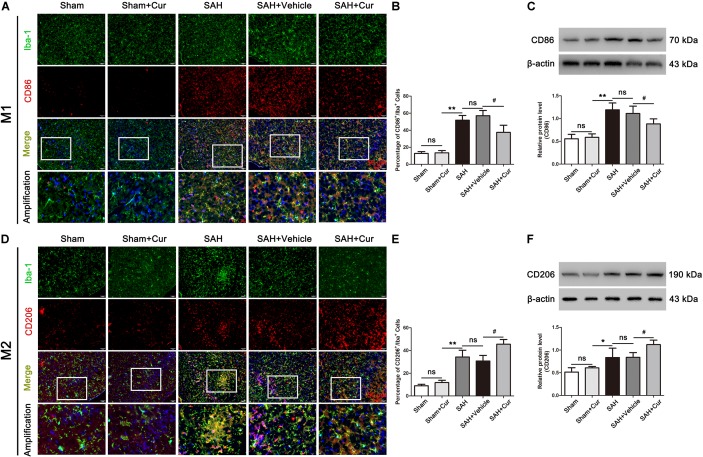
Effects of curcumin treatment on microglia polarization at 24 h after SAH. **(A,D)** Representative immunofluorescence staining images of M1 (Iba-1^+^/CD86^+^, Iba-1 = green, CD86 = red) and M2 (Iba-1^+^/CD206^+^, Iba-1 = green, CD86 = red). Amplification images of phenotype M1 and M2 shown in white box of all groups. **(B,E)** Quantification showed that curcumin treatment reduced the elevated proportion of M1 (Iba-1^+^/CD86^+^), and further elevated the amount of M2 (Iba-1^+^/CD206^+^) at 24 h after SAH compared with the SAH + vehicle group. **(C,F)** Curcumin administration decreased the protein level of CD86 and increased the expression of CD206 as compared with the SAH + vehicle group at 24 h after SAH. The quantitative data are the mean ± SD (*n* = 8, each; ^∗^*p* < 0.05, ^∗∗^*p* < 0.01 vs. Sham + Cur group; ^#^*p* < 0.05 vs. SAH + Vehicle group; ^*ns*^*p* > 0.05). Scale bars = 50 μm.

**FIGURE 6:**
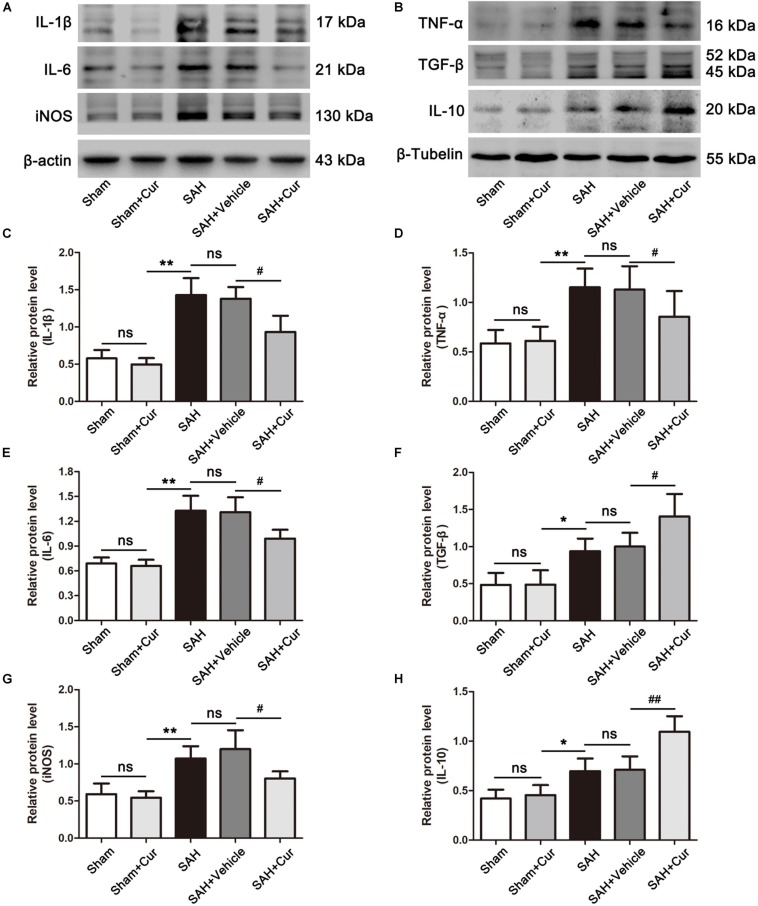
Effects of curcumin treatment on neuro-inflammatory cytokines release at 24 h after SAH. **(A,B)** Western blotting showed curcumin treatment obviously reduced the elevated pro-inflammation cytokines IL-1β **(C)**, IL-6 **(E)**, TNF-α **(D)**, and iNOS **(G)** as compared with the SAH + vehicle group, meanwhile increased the expressions of anti-inflammation mediators TGF-β **(F)** and IL-10 **(H)** at 24 h after SAH. The quantitative data are the mean ± SD (*n* = 5, each; ^∗^*p* < 0.05, ^∗∗^*p* < 0.01 vs. Sham + Cur group; ^#^*p* < 0.05, ^##^*p* < 0.01 vs. SAH + Vehicle group; ^*ns*^*p* > 0.05).

To investigate the role of curcumin on anti-inflammation response and microglia polarization, the protein levels of phenotype M1 markers CD86 and pro-inflammation mediators IL-1β, IL-6, iNOS, and TNF-α, and M2 markers of CD206 and anti-inflammation cytokines IL-10 and TGF-β were measured at 24 after SAH. As shown in Figures 5A,D, CD86^+^/Iba−1^+^ and CD206^+^/Iba−1^+^ represent M1 phenotype and M2 phenotype, respectively. SAH induce significantly increased the proportion of M1 and M2, when compared with the Sham + Cur group. However, curcumin treatment down-regulated the ratio of M1, meanwhile up-regulated the M2 in the temporal cortex as compared with the SAH + vehicle group (Figures 5B,E). To further confirm the efficacy of curcumin on regulation of microglia polarization, western blots analysis was used. The results showed that SAH injury markedly increased the expressions of CD86 (Figure 5C), CD206 (Figure 5F) and inflammation factors IL-1β, IL-6, iNOS, TNF-α, IL-10, and TGF-β relative to the Sham + Cur group (Figures 6A,B). Administration with curcumin significantly reduced the protein levels of CD86 and pro-inflammation cytokines IL-1β, IL-6, iNOS, and TNF-α as compared with the SAH + vehicle group, meanwhile, increased the expressions of CD206 and anti-inflammation cytokines IL-10 and TGF-β (Figures 5, 6). These results were consistent with the immunofluorescence staining.

### Curcumin Treatment Suppressed TLR4 Signaling Pathway Post SAH

**FIGURE 7:**
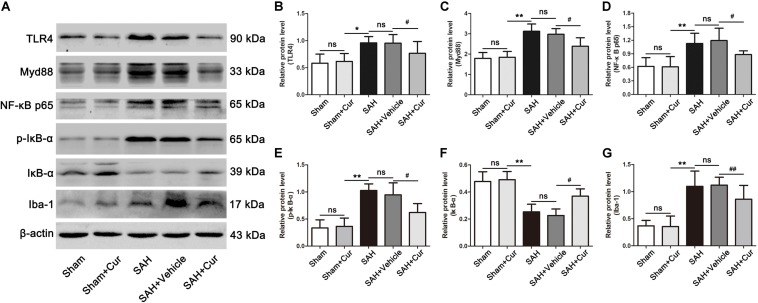
Effects of curcumin on TLR4/Myd88/NF-κB signaling pathway at 24 h after SAH. **(A)** Western blotting showed that administration of curcumin significantly reduced the protein levels of TLR4 **(B)**, Myd88 **(C)**, NF-κB p65 **(D)**, p-IκB-α **(E)**, and Iba-1 **(G)** compared with the SAH + vehicle group. In contrast, curcumin treatment elevated the reduced level of IκB-α **(F)**. The quantitative data are the mean ± SD (*n* = 5, each; ^∗^*p* < 0.05, ^∗∗^*p* < 0.01 vs. Sham + Cur group; ^#^*p* < 0.05, ^##^*p* < 0.01 vs. SAH + Vehicle group; ^*ns*^*p* > 0.05).

The expressions of TLR4 signaling were examined via western blots analysis. Results showed that TLR4, Myd88, NF-κB p65, p-IκB-α, and Iba-1 were dramatically increased in the SAH group and SAH + vehicle group as compared with the Sham + Cur group at 24 h. While these proteins were significantly decreased after curcumin treatment, when compared with the SAH + vehicle group (Figures 7A–E,G). In contrast, the protein level of IκB-α was down-regulated in SAH group and SAH + vehicle group as relative to Sham + Cur group, and curcumin treatment induced its expression when compared with the SAH + vehicle group at 24 h (Figures 7A,F).

### TLR4 Deficiency Promoted Effects of Curcumin on M2 Polarization Post SAH

**FIGURE 8:**
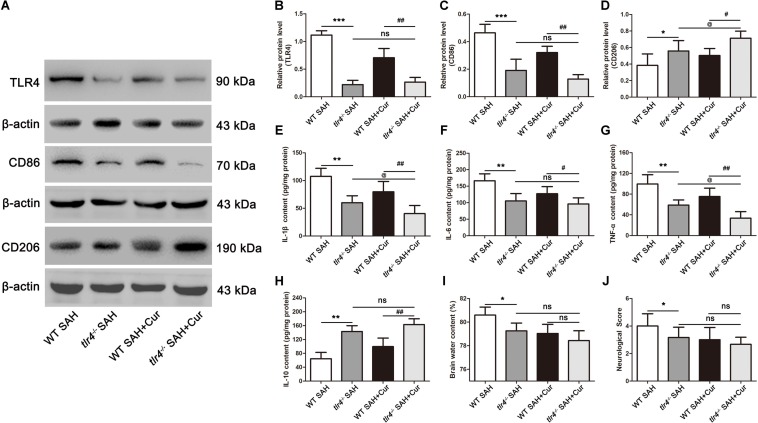
TLR4 deficiency enhanced the effects of curcumin on microglia phenotype switch into M2 at 24 h after SAH. **(A)** Western blotting showed that TLR4 deficiency reduced the protein levels of TLR4 **(B)** and CD86 **(C)** post SAH as compared with the wild type (WT) SAH group, and increased the expression of CD206 **(D)**. Curcumin treatment in *tlr4*^–/–^ group further elevated the level of CD206 as relative to the *tlr4*^–/–^ SAH group and had no statistic difference on TLR4 and CD86. **(E–H)** ELISA showed that the contents of IL-1β **(E)**, IL-6 **(F)**, TNF-α **(G),** and IL-10 **(H)** significantly changed in *tlr4*^–/–^ SAH group compared with the WT SAH group, and curcumin treatment in *tlr4*^–/–^ mice further suppressed the release of pro-inflammation cytokines (IL-1β and TNF-α) and increased the level of IL-10 as relative to the *tlr4*^–/–^ SAH group. **(I,J)** Curcumin treatment in *tlr4*^–/–^ mice had no significant difference on brain water content and neurological score as relative to the *tlr4*^–/–^ SAH group and WT SAH + Cur group. The quantitative data are the mean ± SD (*n* = 9, each; ^∗^*p* < 0.05, ^∗∗^*p* < 0.01, ^∗∗∗^*p* < 0.001 vs. WT SAH group; ^#^*p* < 0.05, ^##^*p* < 0.01 vs. WT SAH + Cur group; ^@^*p* < 0.05 vs. *tlr4*^–/–^ SAH group; ^*ns*^*p* > 0.05).

To further confirm the effect of curcumin on microglia polarization via TLR4 after SAH, we used *tlr4^–/–^* mice to examine the microglia phenotype markers of CD86 and CD206, corresponding to M1 and M2, brain water content and neurological score. As shown in Figures 8A,B, the expression of TLR4 was dramatically decreased in *tlr4^–/–^* mice as compared with the WT mice after SAH at 24 h. Moreover, treatment with curcumin in *tlr4^–/–^* mice significantly reduced the level of TLR4 after SAH, as relative to WT mice in SAH + Cur group, while the expression of TLR4 had no significantly different between *tlr4^–/–^* SAH group and *tlr4^–/–^* SAH + Cur group. Consistent with TLR4, the protein levels of CD86 and pro-inflammation cytokines (IL-1β, IL-6, and TNF-α) were intensely decreased in *tlr4^–/–^* mice when compared with the WT mice after SAH, and curcumin treatment in *tlr4^–/–^* mice suppressed these protein expressions in comparison with WT SAH + Cur mice. Among tlr4^–/–^ SAH group and *tlr4^–/–^* SAH + Cur group, curcumin treatment further decreased these protein levels except CD86 and IL-6 (Figures 8A,C–G). Similarly, compared with the WT mice, TLR4 deficiency effectively increased the expressions of CD206 and anti-inflammation cytokine IL-10 after SAH. Administration of curcumin in *tlr4^–/–^* mice markedly up-regulated the protein levels of CD206 and IL-10 after SAH as compared with the WT SAH + Cur group (Figures 8A,D,H). Curiously, curcumin treatment decreased the brain water content and neurological scores in *tlr4^–/–^* mice relative to WT SAH + Cur group and *tlr4^–/–^* SAH group after SAH at 24 h, while there had no statistic difference between these groups (Figures 8I,J).

## Discussion

Many researches have indicated inflammation participation in EBI after SAH. Microglia, as the resident macrophages in central nervous system (CNS), play a key role in modulation of neuro-inflammation in the pathogenesis of EBI (Pang et al., 2018). In this study, *tlr4*^–/^*^–^* mice were used to investigate the effect of TLR4 on the polarization of microglia, and observed that TLR4 deficiency inhibit the expressions of M1 cytokines including IL-1β, IL-6, and TNF-α, and promote microglial polarization toward M2 phenotype and the release of anti-inflammation mediator IL-10. Meanwhile, we assessed the potential neuroprotective effects of curcumin on microglia-induced inflammation and explored the possible underlying mechanism on microglia polarization following SAH. We observed that administration of curcumin decreased the proportion of M1 relative to M2 and pro-inflammatory cytokines through inhibiting the TLR4/Myd88/NF-κB signaling pathway, and curcumin treatment in *tlr4*^–/^*^–^* mice further promote microglia phenotype shift toward M2 after SAH. Thus far, the present study is the first time to investigate the possible molecular mechanism of curcumin on microglia-inflammation and microglial polarization following SAH.

Numerous studies showed that neuro-inflammation became emerged an important player in the progression of pathophysiology of SAH, which includes activation of resident microglia and recruitment of immune cells such as neutrophils from the blood, as well as amount of production of pro-inflammation mediators (van Gijn et al., 2007; Fujii et al., 2013; de Oliveira Manoel and Macdonald, 2018). Recently, researchers discovered that microglia with different phenotypes has distinct effects on the prognosis of stroke patients (Hu et al., 2012, 2015). In the early stage of pathological injury of SAH, M1 dominated microglia activation parallels with extreme inflammation reaction and aggravated brain damage (Girard et al., 2013; Liu et al., 2017). In agreement with this theory, our results found that SAH induced functional markers of activation M1 (IL-1β, IL-6, and TNF-α) significantly increased, which leading to exacerbated brain edema, neuronal apoptosis, and neurological dysfunction in EBI.

Previous studies demonstrated that curcumin may possess pleiotropic activities in a variety of stroke-induced brain damage, exhibited anti-oxidative, anti-inflammation, regulation neuronal apoptosis, and anti-tumor properties (Thiyagarajan and Sharma, 2004; Zhao et al., 2010; Wang et al., 2016; Xu et al., 2016; Maiti et al., 2019). Among these neuroprotective functions, curcumin-mediated anti-inflammation exhibited promising therapeutic benefits. Zhu et al. (2014) showed that curcumin intraperitoneal injection could inhibit neuro-inflammation cascade and alleviate the inflammation injury after TBI. This neuroprotection was mediated through reducing activation of TLR4 receptor signaling pathway. In the mice model of ischemic cerebral injury, administration of curcumin reduced the infarct volume and improved functional outcomes through regulatory microglia polarization and promoting its shift to M2 (Liu et al., 2017). However, the effects of curcumin on modulation microglia polarization to intervene inflammation response have not been explored in SAH. Consistent with this studies, we found that administration of curcumin could significantly inhibit cerebral inflammation and pro-inflammation cytokines (IL-1β, IL-6, and TNF-α) release, meanwhile up-regulate anti-inflammatory medium (IL-10) after SAH. Notably, our results showed that treatment with curcumin could induce microglia polarization and shift pro-inflammation phenotype M1 toward anti-inflammation phenotype M2 in the cerebral cortex at 24 h following SAH. Moreover, curcumin treatment improved neurological function outcomes and ameliorated brain edema, as well as neuronal apoptosis, which might through regulating M2 microglia polarization. While the potential mechanisms of curcumin on microglia polarization after SAH are poorly understand.

How did curcumin regulate the microglia phenotype after SAH? As we all known TLR4, pivotal member receptor, is highly expressed in microglia cells, and which play an important role in regulating neuro-inflammatory response and innate immunity (Kanazawa et al., 2017). Furthermore, growing evidences indicated that TLR4 signaling pathway played a critical role on microglia polarization (Rosciszewski et al., 2018). Yang et al. demonstrated that TLR4 mediated MyD88/NF-κB signaling pathway was involved in microglia polarization, and inhibiting this pathway contributed to conversion of microglia to M2 phenotype in hippocampus of a TBI rat model (Yang et al., 2019). In BV-2 microglia cells, Huang et al. found that microglia activated toward M1 phenotype and induced release of pro-inflammation mediators upon neurotoxicant exposure, which might be through activation TLR4/MyD88/NF-κB pathway (Huang et al., 2019). Moreover, Yao et al. showed that TLR4 deficiency promoted microglia phenotype switch to M2 and provided neuroprotection after TBI (Yao et al., 2017). Regarding the relationship between curcumin and TLR4 pathway, multiple evidence suggests that curcumin inhibited neuro-inflammation through down-regulation TLR4/MyD88/NF-κB pathway. Kong et al. demonstrated that curcumin reduced the formation of NLRP3 inflammasome and secretion of pro-inflammation mediators via TLR4 signaling cascade in macrophages (Kong et al., 2016). *In vitro* and *in vivo* of TBI models, curcumin suppressed microglia activation and inflammation response through inhibition of TLR4 pathway (Zhu et al., 2014). With these cumulative studies, herein we speculated that curcumin promoted M2 polarization to reduce neuro-inflammation following SAH, which might depend on inhibition of TLR4/MyD88/NF-κB pathway.

In our experiment, our result showed that the expression of TLR4 was significantly increased at 24 h after SAH. Then treated with curcumin, due to its characteristics short serum half-life and penetrating the blood-brain barrier, we chose to maximize its bioavailability via intraperitoneal injection at 15 min following SAH depending on previous publications. The data revealed that curcumin treatment dramatically inhibited expression of TLR4, MyD88, NF-κB p65 and phosphorylation IκB-α 24 h after SAH, induced microglia phenotype switch to M2, and suppressed pro-inflammatory release. Considering these results, we suggested that curcumin promote M2 polarization to against neuro-inflammation after SAH might via regulating TLR4 pathway cascade. Upon further confirm this speculation, TLR4 KO mice was used and results showed that TLR4 deficiency improved neurological function and induced microglia phenotype shift to M2, as well as inhibited release of M1 mediators compared with the WT mice at 24 h after SAH. Therefore, we concluded that curcumin provided neuroprotection via regulation of microglia polarization after SAH, which might through modulation TLR4/MyD88/NF-κB signaling pathway.

In conclusion, our experiment demonstrate that curcumin could reduce neuro-inflammation response, which protects against SAH via shifting microglia phenotype toward M2, to attenuate secretion of pro-inflammation mediators and improve neurological deficits. The absence of TLR4 further confirmed that the effect of curcumin on promoting M2 polarization might through inhibiting the TLR4/MyD88/NF-κB signaling pathway. Thus, curcumin would be a potential therapeutic agent to alleviate inflammation response in EBI after SAH.

## Data Availability Statement

All datasets generated for this study are included in the article/supplementary material.

## Ethics Statement

The animal study was reviewed and approved by the Institutional Animal Care and Use Committee at Nanjing Drum Tower Hospital and the National Institutes of Health (NIH) Guide for the Care and Use of Laboratory Animals.

## Author Contributions

YG designed the project and wrote the manuscript. ZZ participated in the SAH model and analyzed the data of the animal studies. YL performed the ELISA. TT, YZ, and GL performed the immunofluorescence staining and TUNEL staining. HW, DZ, and LW performed the Western blotting. HD analyzed the samples and data. WL and CH contributed to conceive and provided critical revisions. All authors checked and approved the final manuscript.

## Conflict of Interest

The authors declare that the research was conducted in the absence of any commercial or financial relationships that could be construed as a potential conflict of interest.
